# Investigating the renoprotective effect of C21 in male mice with sepsis via modulation of p-AKT/PI3K expression

**DOI:** 10.25122/jml-2022-0299

**Published:** 2023-02

**Authors:** Huda Jabber, Bassim Mohammed, Najah Rayish Hadi

**Affiliations:** 1 Department of Pharmacology and Therapeutics, College of Medicine, University of Al-Qadisiyah, Iraq; 2 Department of Pharmacology and Therapeutics, Faculty of Medicine, University of Kufa, Kufa, Iraq

**Keywords:** C21, Acute kidney injury, sepsis, mice, P-AKT/PI3K, ACR – Urinary Albumin and Creatinine, AKT – Acute Kidney Injury, BUN – Blood Urea Nitrogen, CDC – Centers for Disease Control and Prevention, CLP – Cecal Ligation Procedure, IHC – Immunohistochemistry, P-AKT – Phosphorylated Protein kinase B, PCR – Polymerase Chain Reaction, PI3K – Phosphatidylinositol-3-kinase, RAS – Renin-Angiotensin System, SCR – Serum Creatinine, UF – Urine Flow, UNaV – Urinary Na Volume

## Abstract

This study aimed to investigate if C21 could prevent acute renal injury induced by sepsis by regulating the expression of p-AKT/PI3K. Five equal groups of 25 adult male Swiss-albino mice were randomly divided (n=5): sham (laparotomy without CLP), CLP, vehicle (equivalent amount of DMSO one hour before CLP), and C21 (0.03 mg/kg, one hour before CLP). ELISA was used to measure serum inflammatory mediators, and the expression of PI3K and P-AKT was determined using PCR and immunohistochemistry (IHC), respectively. TNF, TNF receptor, F8-isoprostane, urea, creatinine, and IL-6 blood levels were considerably lower in the CLP group (p<0.05) compared to the sham group, whereas the C21 treated group had significantly (p<0.05) greater levels of these inflammatory mediators. The IHC analysis revealed that P-AKT expression was significantly lower (p<0.05) in the CLP group compared to the sham group, while the C21 pretreatment group had significantly higher levels of P-AKT expression compared to the CLP group (p<0.05). The PI3K expression in the CLP group was significantly lower than in the sham group (p<0.05), according to PCR results, whereas the PI3K expression in the C21 pretreatment group was significantly greater than in the CLP group (p<0.05). This study showed that C21 might reduce levels of pro-inflammatory cytokines, including TNF-, IL-6, and TNF receptor, by modulating the PI3K/AKT signaling pathways, which can, in turn, reduce renal dysfunction during CLP-induced sepsis in male mice.

## INTRODUCTION

According to the Centers for Disease Control and Prevention (CDC), sepsis is a severe, life-threatening systemic inflammatory reaction to infection that develops when the immune system releases chemicals into the bloodstream, causing an inflammatory state crucial to overcoming infections [1]. There are several risk factors for developing sepsis-induced acute kidney injury (AKI), such as age, chronic disorders, weakened immune systems, and medical devices [2]. Each year, sepsis causes more than 1.5 million cases and kills more than 250,000 Americans. Sepsis, severe sepsis, and septic shock may be distinguished by their symptoms, and the earlier they are treated, the better the prognosis [3]. Sepsis is a generalized severe inflammatory reaction brought on by a breakdown in the host's ability to fight off infection, which can affect several organs, including the kidneys [4]. Major surgery, heart failure, respiratory failure, hypovolemia, and other conditions linked to shock and poor perfusion are additional causes of AKI. The primary cause of sepsis-induced AKI is tissue hypoperfusion. Resuscitation that aims to improve tissue perfusion is thought to lessen or even reverse kidney damage [5]. In general, sepsis affects the kidneys, which are also at a high risk of dying. Sepsis-induced AKI is complicated and multifactorial, causing endothelial dysfunction, intra-renal hemodynamic changes, inflammatory cell infiltration in the renal parenchyma, obstruction of tubules with necrotic cells and debris, and intra-glomerular thrombosis. Innate immunity is triggered when host-microbe interaction starts, and coordinated cellular and humeral defense responses result in the release of cytokines (IL-1, IL-18, IL-6, and TNF-alpha), which create a cytokine storm state and hemodynamic instability that cause organ dysfunction, septic shock, and death. This pro-inflammatory response is offset by a compensatory anti-inflammatory response characterized by altered cytokine production, altered monocyte antigen presentation, reduced lymphocyte proliferation, and hastened apoptosis [6]. Blood pressure homeostasis has long been attributed to the renin-angiotensin system (RAS), which has received much attention in the field of cardiovascular physiology. Proliferation, fibrosis, and inflammation are among additional consequences that have later been mentioned [7]. The AT1 and the AT2 receptors, which are the RAS's two primary receptors, both play a complex dual function in inflammation. Uncontrolled inflammation is known to induce tissue damage through the AT1 receptor, while the AT2 receptor is thought to have positive and protective effects. C21 (Compound 21), derived from the prototype nonselective AT(1)/AT(2) receptor agonist L-162, 313 is the first non-peptide selective angiotensin II type 2 receptor (AT2R) agonist. Furthermore, to date, there have been no reports focusing on the medicinal chemistry perspective of peptide AT2R agonists [8].

## MATERIAL AND METHODS

For this experimental study, 25 adult male mice (Swiss albino) aged 8–12 weeks and weighing 20-30g were obtained from the animal house of the College of Science at Al-Kufa University. The mice were housed in a controlled environment with a temperature of 25±2°C, humidity at 60–65%, and a 12 h dark/12 h light cycle. They were kept in isolated cages with free access to water and a standard diet until the start of the investigation at the animal house of Al-Kufa Medical College.

This study followed the guidelines of the Association for Laboratory Animal Science's Guide for the Care and Use of Laboratory Animals, and the Animal Care Committee of Al-Kufa University approved all procedures related to animal care and welfare. Furthermore, all efforts were made to minimize the discomfort and suffering of the mice, which were anesthetized with ketamine and xylazine during the surgical procedures. After two weeks of acclimatization, the mice were randomly divided into five groups:

- Sham group (negative control): mice underwent the same anesthetic and surgical procedures (laparotomy) for an identical period for the sepsis-induced procedure without cecal ligation procedure (CLP) induction;
- CLP group: all mice underwent the CLP procedure;
- Vehicle group: mice received DMSO 10 % (v/v) as a standard vehicle;
- Control group (positive control): CLP induction and no treatment;
- C21 treated CLP group: all mice received C21 (0.03 mg/Kg) intra-peritoneal injection 1 hour before the CLP procedure [7].

Finally, identical doses of the vehicle were administered to the pretreated animals in the CLP group simultaneously, for the same length of time, and in the same manner as the vehicle pretreated group [8]. The CLP model was chosen for its remarkable similarity to actual sepsis, as it involves infection with a diverse microbial flora from the animal itself via contact with devitalized tissue. When comparing research, variables in surgical techniques and postoperative care should also be considered. For instance, the position of the suture, the size of the needle, and the number of punctures may have a significant impact on the amount of pro-inflammatory cytokines released into the peritoneum and serum as well as the development of the sickness. The cecal ligation and piercing paradigm were used to induce polymicrobial sepsis, and all mice were given anesthesia via ketamine (100 mg/kg) and xylazine (10 mg). The abdominal laparotomy was performed after shaving a 1.5 cm midline incision, and the cecum was exposed to prevent eye dryness. Ophthalmic ointment was applied to the eyes throughout the procedure. After that, a G-20 double puncture was performed after ligating the cecum immediately below the ileocecal valve. C21 was purchased from Medchem Express MCE in the USA and made in dimethyl sulfoxide (DMSO) as the standard vehicle before being diluted in N.S. at a ratio of 1:1 and prepared just before usage. The medication was given intraperitoneally in a dosage of 0.03 mg/Kg, weight-based, one hour before the CLP procedure [9]. The histological alterations in the cortex and exterior stripe of the external medulla (OSOM) were evaluated using quantitative assessments of tissue damage by a blind observer. The tubular damage symptoms identified included tubular epithelial enlargement, loss of brush boundary, vacuolar degeneration, necrotic tubules, cast development, and desquamation. The histological sections of all groups were inspected and graded following Zingarelli's technique [10].

## RESULTS

**Figure 1:**
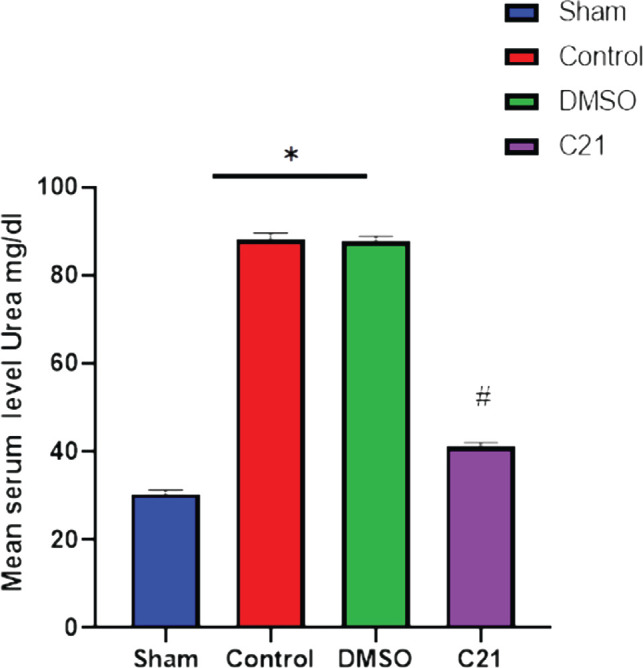
Comparison of median urea levels (mg/dL) in the four experimental groups 24 hours after sepsis induction. Statistical significance: A: P<0.05 compared to the corresponding sham group; B: P<0.05 compared to the sepsis group. Data presented as mean ± standard error of the mean.

**Figure 2:**
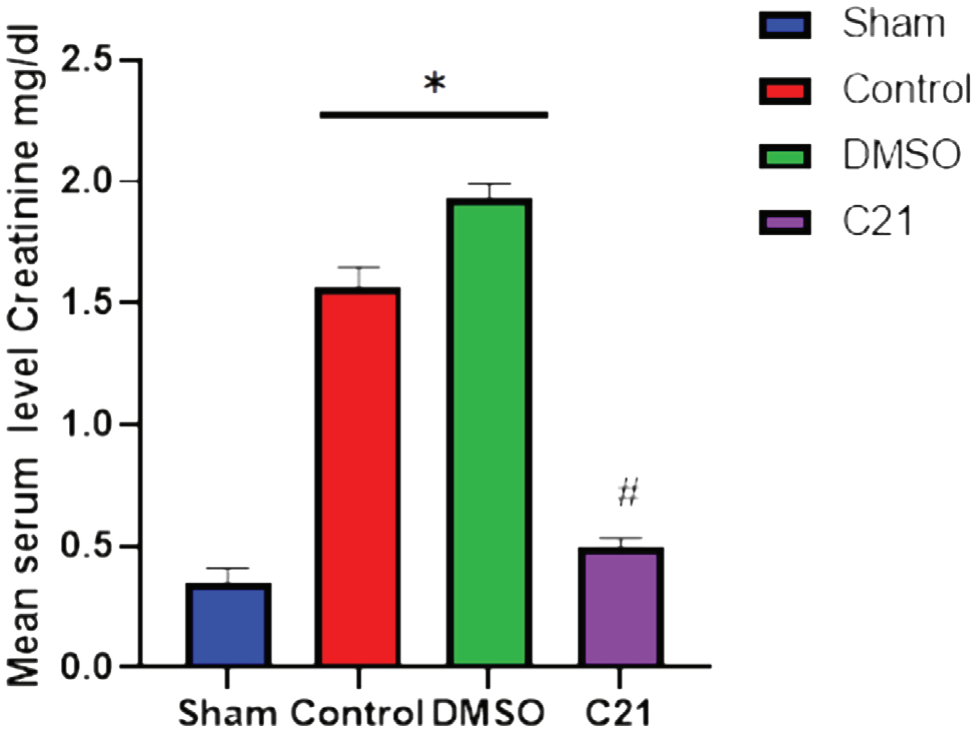
Comparison of median creatinine levels (mg/dl) 24 hours after sepsis induction in the four experimental groups. Data presented as mean±standard deviation. A: P<0.05 vs. the matching sham; B: P<0.05 vs. sepsis mice.

The impact of therapy on renal function was evaluated 24 hours after sepsis induction by assessing urea and creatinine levels. Compared to the sham group, blood levels of urea and creatinine were significantly higher in the sepsis and vehicle groups (p<0.05), whereas the C21 pretreatment group had significantly lower blood levels of urea and creatinine than the CLP group (p<0.05), as shown in Figures 1 and 2.

**Figure 3:**
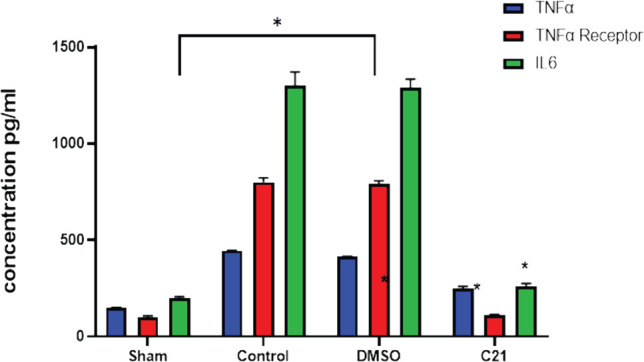
Levels of pro-inflammatory cytokines (TNF-α, TNF-α receptor, and IL-6) in blood and renal tissue of the four experimental groups 24 hours after sepsis induction. The data are presented as mean±SEM; *P<0.05 versus the sham group; P<0.05 versus the sepsis group; and ^#^P<0.05 compared to sepsis-treated mice.

The sepsis and vehicle groups had significantly higher amounts of pro-inflammatory cytokines (TNF-A, TNF-receptor, and IL-6) in both blood and renal tissue compared to the control group (p<0.05). In contrast, the C21 pretreatment group had significantly lower levels of pro-inflammatory cytokines in blood and renal tissue compared to the CLP group (p<0.05), as shown in Figure 3.

**Figure 4:**
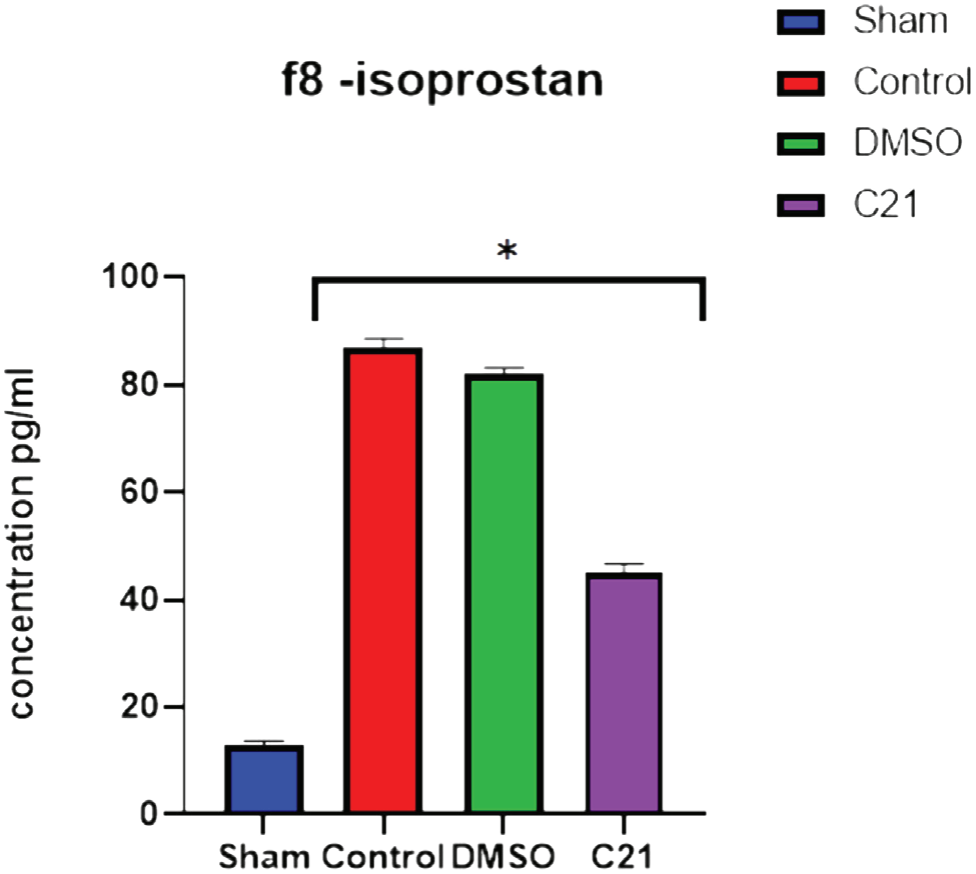
Tissue levels of F8-isoprostane (pg/ml) in the four experimental groups 24 hours after sepsis induction by the CLP model. The data are presented as mean SEM; *P 0.05 compared to equivalent sham; P 0.05 compared to sepsis mice; and ^#^P 0.05 compared to sepsis mice treated.

To obtain more detailed findings, ELISA was used to determine the tissue levels of the specific damage marker F8-Isoprostan in all experimental groups 24 hours after CLP-induced polymicrobial sepsis. ELISA outcomes demonstrated that the sepsis and vehicle groups had significantly higher tissue levels compared to the sham group (p<0.05), while the C21 pretreated group had significantly lower levels of F8-Isoprostane as compared with the untreated sepsis group (p <0.05) (Figure 4).

**Figure 5:**
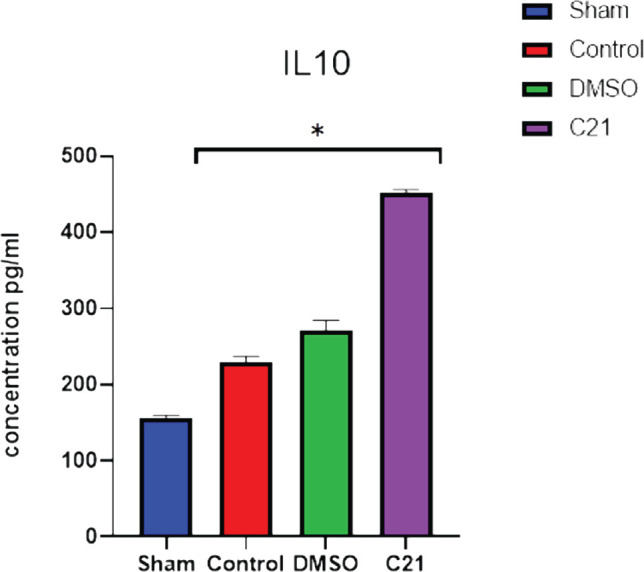
Blood IL-10 levels (pg/ml) in the four experimental groups 24 hours after sepsis induction: Data presented as mean SEM; *P<0.05 vs. corresponding sham group; P<0.05 vs. sepsis group; ^#^P<0.05 vs. sepsis mice treated.

The analysis of the data revealed that, as compared to the sham group, the sepsis and vehicle groups had significantly (p<0.05) greater blood levels of IL-10, while serum IL-10 levels in the C21 pretreatment groups were significantly (p<0.05) greater than those in the sepsis group (Figure 5).

**Figure 6:**
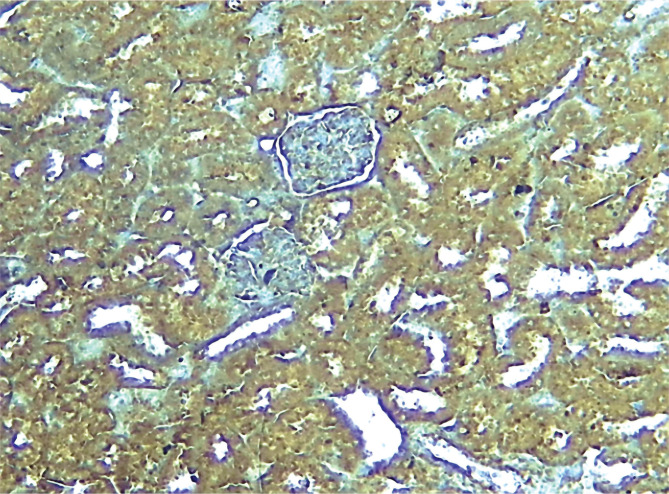
Immunostain kidney segment from the control group in photomicrograph demonstrates positive P-AKT (brown hue) x 100.

**Figure 7:**
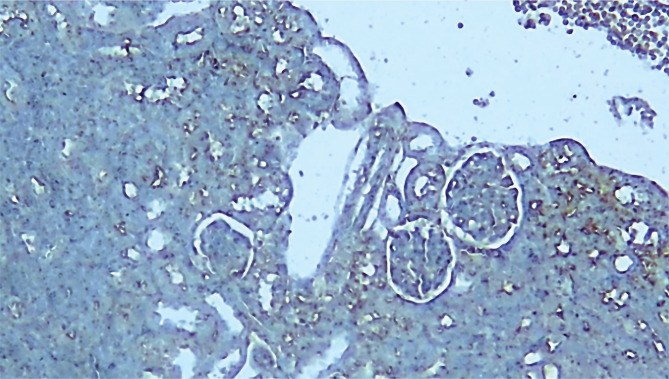
Photomicrograph of immune-stained kidney section of control sepsis group shows negative P-AKT (blue color) x100.

**Figure 8:**
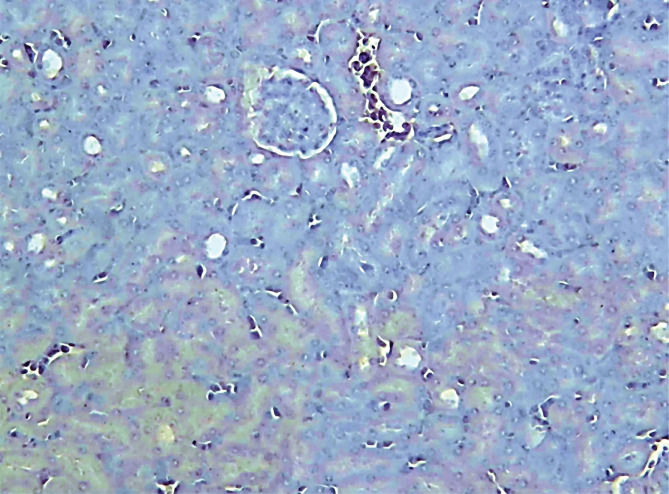
Negative P-AKT (blue color) x100 is seen in the photomicrograph of the immune-stained kidney segment of the DMSO-sepsis group.

**Figure 9:**
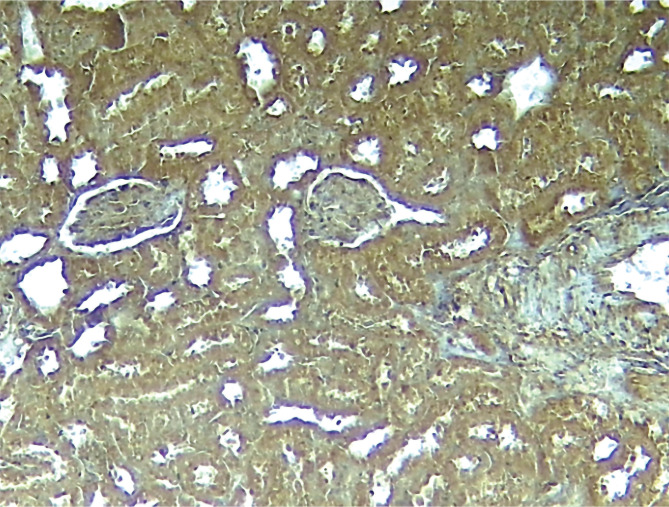
Strongly significant P-AKT (brown color) x100 is shown in the photomicrograph of immune-stained kidney tissue from the sepsis group treated with C21.

**Figure 10:**
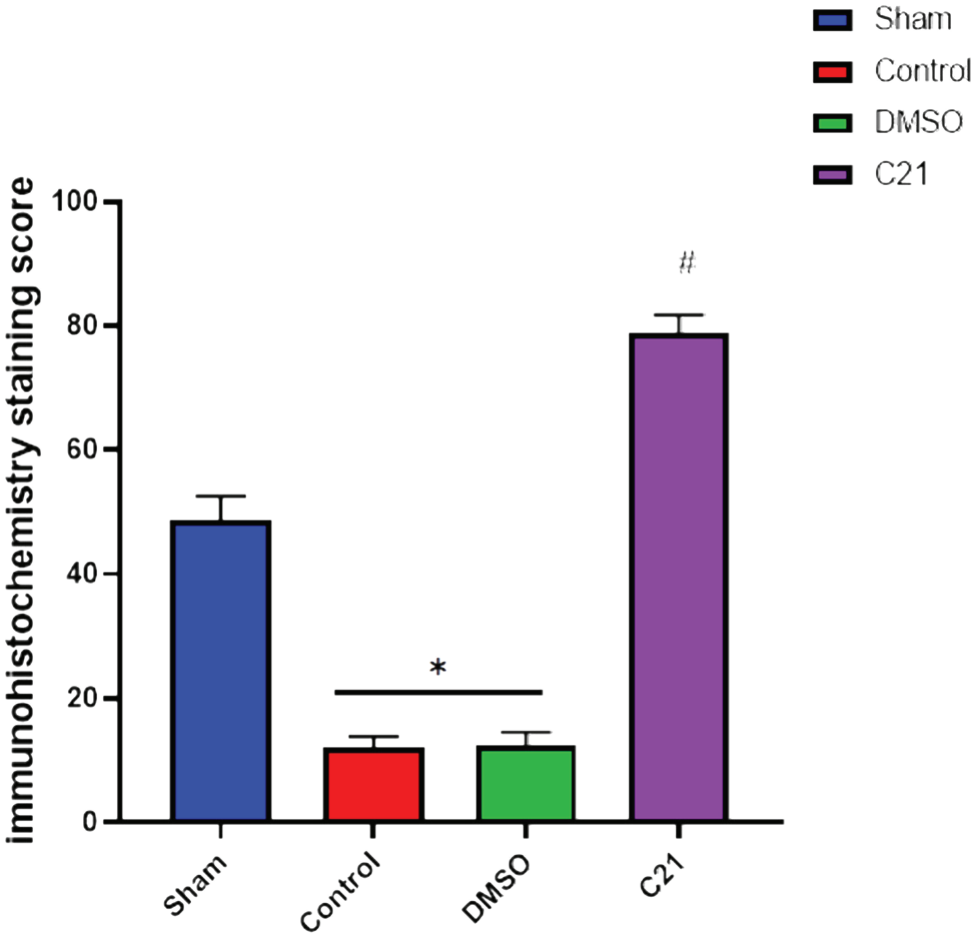
Renal tissue P-AKT staining scores of all groups measured by IHC; C21-treated group shows significantly improved P-AKT results compared to the sepsis control group (#P 0.05 vs. control).

The immunohistochemistry results of the current study showed that the P-AKT interaction was visible by brown cytoplasmic staining in sections of the pretreated group C21. The sections from the control and vehicle groups showed no cytoplasmic staining, unlike the pretreated groups. All the above results are summarized in Figures 6–10.

**Figure 11:**
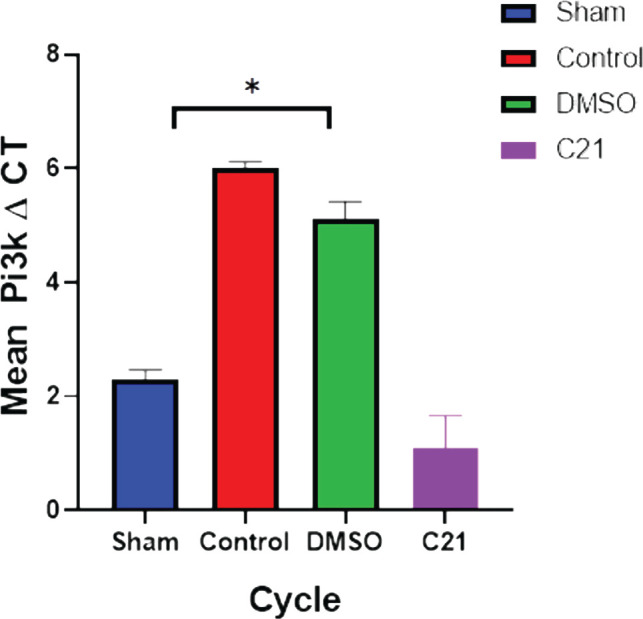
The average Pi3k CT values 24 hours after sepsis. The data are presented as mean SEM; * P 0.05 vs. matching sham; P 0.05 versus sepsis mice; and ^#^P 0.05 versus sepsis mice treated. A lower CT value denotes increased gene expression.

The effects of C21 therapy on the intracellular signaling system were also investigated by PCR analysis, which focused on regulating the effects of the medication on PI3K signaling cascades during CLP-induced polymicrobial sepsis. Compared to the sham group, the expression of the PI3K signaling cascades in renal cells was considerably lower in the sepsis and vehicle groups (p<0.05), while it was significantly greater in the C21 pretreatment groups (p<0.05), as shown in Figure 11.

## DISCUSSION

One adverse effect of sepsis, characterized by a severe inflammatory response to infection, is acute kidney injury. Accurate animal models representing human disease are crucial because they speed up the development of treatments that can be used in clinical settings. However, animal models often do not accurately reflect human disease. The first line of sepsis therapy involves medicines and volume resuscitation. The discovery of antibiotics has significantly reduced the morbidity and mortality of infectious illnesses during the last decades.

Nevertheless, excessive inflammatory response and immune function decline cause numerous organ injuries that affect clinical outcomes [11]. To the best of our knowledge, no studies have evaluated how C21 affects renal function in sepsis-induced renal damage. However, it has been suggested that C21 decreased glomerular filtration rate and significantly decreased immune cell infiltration in the interstitial space in HSD-fed rats, which may have contributed to decreased tubulointerstitial fibrosis [12]. C21 has been shown to increase urinary Na volume and flow (UF) (UNaV) by activating AT2 receptors in obese Zucker rats, resulting in natriuresis [12].

In this investigation, the sepsis group considerably outperformed the sham group in terms of blood levels of TNF-, TNF- receptor, and IL-6 (P<0.05). The sepsis-induced renal damage led to an increase in TNF, TNF receptor, and IL-6 levels. One study demonstrated that TNF-α levels were elevated 24 hours after sepsis induction in mice [13]. Inosine monophosphate decreased tumor necrosis factor (TNF)-production and increased IL-10 production in endotoxemic mice [14]. In this study, the level of F8- isoprostane was significantly elevated in the sepsis group compared to the sham group (P <0.05). Stem cell-derived exosomes have been shown to significantly reduce the levels of blood urea nitrogen (BUN), serum creatinine (SCR), urine albumin and creatinine (ACR), and 8-isoprostane associated with renal failure [15]. The AT2R antagonist PD dramatically reduced the rise in IL-10 levels caused by treatment with C21 in both the plasma and kidney, pointing to the involvement of AT2R [16]. Lean Zucker rats were given C21, which increased IL-10 levels in the plasma and renal cortex by a factor of three. This increase was visible even in the absence of LPS.

AKT, also known as protein kinase B, is a serine/threonine-specific protein kinase essential for many physiological functions. Once active, AKT controls a wide range of proteins involved in cell growth, proliferation, motility, adhesion, neovascularization, and cell death by activating or suppressing their activity via phosphorylation. When compared to the sham group, the control and vehicle groups in the current study had considerably lower levels of P-AKT, and these findings were consistent with a previous study [17], which showed that lipopolysaccharide (LPS) lowered phosphorylated protein kinase B (p-AKT) at the molecular level in research on sepsis. In another study, the LPS group had lower expression levels of the phosphorylated forms of the p-PI3K, p-AKT, and p-mTOR proteins [18,19].

Compared to the control and vehicle control groups, the level of the pro-inflammatory cytokines (TNF-, TNF-receptor, and IL-6) was considerably lower in the C21-pretreated group (P<0.05). C21 generated a substantial decrease of these inflammatory factors in a mouse model of cutaneous inflammation, where IL-6, MCP-1, and TNF- mRNA were upregulated [20]. Compared to the control and vehicle groups, the amount of F8-Isoprostane in renal tissue was considerably lower (P<0.05) in the C21 pretreatment group. In previous studies, continuous therapy with the AT2R agonist C21 has been shown to have a protective effect on diabetes by regulating oxidative stress and reducing the inflammatory response [21]. In the current study, C21 significantly increased tissue P-AKT expression (P<0.05) compared to the sepsis group, and to the best of our knowledge, no studies have evaluated the impact of C21 on P-AKT in sepsis-induced kidney damage. In other studies, C21 has been shown to increase AKT phosphorylation in skeletal muscle, and in this study, C21 caused significantly higher tissue PI3K expression compared to the sepsis group [20]. To the best of our knowledge, no studies have evaluated the impact of C21 on PI3K expression in sepsis-induced kidney damage. However, activating AT2R has been shown to promote in vitro angiogenesis in human uterine artery endothelial cells during pregnancy [22].

## CONCLUSIONS

This study has demonstrated that treatment with C21 can attenuate sepsis-induced renal injury by up-regulating both PI3K expression and P-AKT phosphorylation. This up-regulation was closely related to the suppression of pro-inflammatory mediators such as TNF-α, TNF-α receptor, F8 isoprostane, and IL-6, which are known to reduce renal function. The results suggest that both PI3K and P-AKT could serve as biomarkers and therapeutic targets in patients with sepsis to improve renal function. The RT-PCR and IHC techniques used in this study have shown that C21 pretreatment resulted in higher levels of both PI3K and P-AKT compared to untreated or vehicle mice, indicating the involvement of both PI3K and P-AKT in the renoprotective mechanism of C21 during polymicrobial sepsis. These findings provide new insights into the mechanisms underlying sepsis-induced renal injury and offer potential avenues for developing new treatments for this condition.
